# Changes in T1 slope and cervical sagittal vertical axis correlate to improved neurological function recovery after cervical laminoplasty

**DOI:** 10.3389/fsurg.2022.1002848

**Published:** 2022-09-16

**Authors:** Dong-Fan Wang, Xiang-Yu Li, Chao Kong, Cheng-Xin Liu, Bin Shi, Shi-Bao Lu

**Affiliations:** ^1^ Department of Orthopedics, Xuanwu Hospital, Capital Medical University, Beijing, China; ^2^ National Clinical Research Center for Geriatric Diseases, Xuanwu Hospital, Capital Medical University, Beijing, China

**Keywords:** cervical laminoplasty, T1 slope, clinical outcomes, compensation mechanism, sagittal balance

## Abstract

**Purpose:**

To investigate the influence of changes in T1 slope (T1S) and cervical sagittal vertical axis (CSVA) on cervical laminoplasty outcomes.

**Methods:**

Eighty-one patients with cervical spondylotic myelopathy (CSM) treated with cervical laminoplasty were enrolled in this study. Demographic parameters included age and follow-up time. Imaging data included occiput-C2 lordosis (OC2), C2–C7 Cobb angle (CL), T1S, CSVA. Outcome assessment indicators included the Japanese Orthopedic Association (JOA) score, JOA recovery rate, and neck disability index (NDI). All patients were grouped based on preoperative T1S and variation in CL after surgery, respectively. Patients with decreased CL postoperatively were further grouped according to whether they were combined with T1S reduction.

**Results:**

There were no significant differences in the final JOA score, JOA recovery rate, or NDI between patients with different T1S. Patients with loss of CL postoperatively had lower JOA score and JOA recovery rate, but higher NDI than patients with sustained CL. Furthermore, patients with CL loss but compensate for it with reduction in T1S had lower CSVA, higher JOA score and JOA recovery rate than those with CL loss alone.

**Conclusions:**

Decreased T1S postoperatively prevents the tendency of the cervical spine to tilt forward by regulating CSVA and facilitates recovery of neurological function after cervical laminoplasty.

## Introduction

Cervical spondylotic myelopathy (CSM) results from the nearly universal process of degeneration of the discs and joints of the cervical spine, which has been one of the most common causes of acquired spinal cord dysfunction, including paresthesia, motor weakness, gait disturbance, neck pain/radicular arm pain, hyperreflexia, even bowel/bladder dysfunction (1, 2). Posterior expansive open-door laminoplasty (EOLP) is a mature procedure for halting neurological function deterioration and improving the quality of life for patients with CSM who are unresponsive to conservative treatment (3). This technique reduces intramedullary pressure by allowing the cervical spinal cord to shift backwards through posterior decompression (4). The most significant advantage of EOLP is that it can be applied to multi-level compression cases and preserve the posterior stabilizing elements simultaneously (5). However, there are still possible postoperative complications such as axial pain, decreased range of motion, and loss of lordosis (5).

In recent years, the roles of spinal sagittal parameters in predicting outcomes and neurological function recovery after cervical surgery have become a focus of attention. Research by Chen et al. revealed preoperative cervical sagittal vertical axis (CSVA) was closely associated with neck pain in CSM patients treated by laminoplasty and proposed a cut-off value of the CSVA was 28.9 mm degreed with visual analogue scale >4 (6). In a retrospective study contained 64 patients who underwent cervical laminoplasty for cervical ossification of the posterior longitudinal ligament, Kim et al. demonstrated patients with higher preoperative T1 slope (T1S) had more loss of cervical lordosis (CL) after surgery and might predispose to worse clinical outcomes (7).

However, univariate analyses of the correlation between preoperative sagittal parameters and clinical outcomes are incomprehensive as the cervical and adjacent segments may change simultaneously after surgery to maintain sagittal balance and horizontal gaze (8). Therefore, figuring out the impact of variation in cervical sagittal parameters on suboptimal surgical outcomes after cervical laminoplasty could serve as a significant reference for clinical practice. We present the following hypotheses: (1) preoperative T1S is uncorrelated with postoperative clinical outcomes and (2) the reduction of T1S after cervical laminoplasty is a compensatory mechanism of loss of cervical lordosis (CL) and can halt CSVA tilting forward, which may contribute to the improvement of clinical outcomes. We conduct the present study with the following aims: (1) to measure changes in T1S and CSVA after EOLP and (2) to investigate how variations in T1S and CSVA affect clinical outcomes after cervical laminoplasty.

## Materials and methods

### Patient population

After being approved by the Ethics Committee of Capital Medical University Xuanwu Hospital (approval number: 2018014), a retrospective review of patients who underwent cervical laminoplasty between February 2018 and October 2020 was performed. The inclusion criteria were: (1) age >18 years; (2) clinical presentations indicating cervical spinal cord compression; (3) imaging and neuroelectrophysiological examinations revealing developmental cervical spinal stenosis, multilevel cervical disk herniation, or ossification of the posterior longitudinal ligament; (4) treated by EOLP; and (5) follow-up for at least 12 months. The exclusion criteria were: (1) history of other spine surgery; (2) combined with tumors, tuberculosis, or trauma; and (3) incomplete follow-up or imaging data. A total of 81 patients were eligible eventually.

### Groups

**Figure 1:**
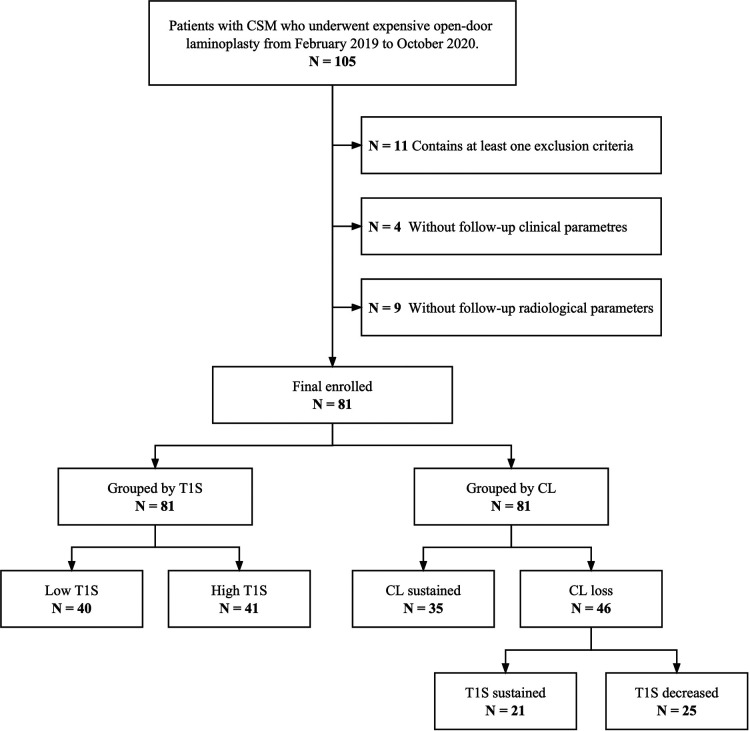
Flowchart of this study.

All patients were grouped according to a median preoperative T1S to assess the correlation between T1S and clinical outcomes. For probing variations in sagittal parameters after surgery, patients were divided into the CL sustained group and the CL loss group based on whether they were complicated with loss of CL after laminoplasty. Furthermore, patients with postoperative CL decreasing were further grouped into the T1S sustained subgroup and the T1S decreased subgroup to investigate the compensatory mechanism of T1S to cervical sagittal malalignment. Figure 1 illustrates the flow chart of this study.

### Surgical procedures

The surgical procedure was performed based on the Hirabayashi method (9) with some modifications. The patient was placed in the prone position with an upward cranial angle of 15–20°. A Mayfield skull clamp was used to immobilize the head. An incision was made on the posterior midline of the cervical spine. The spinous process, lamina, and bilateral lateral mass were exposed. Some of the spinous processes were removed using a rongeur. The paraspinal muscle of C2, especially the semispinalis, was preserved. A high-speed drill was used to create gutters on the bilateral laminae at the border of the laminae and facets. The lamina of the side with more significant clinical symptoms was completely severed and used as the open side. The other side of the lamina was partially cut, with the ventral cortex preserved to form the hinge side. A thin-bladed Kerrison rongeur was used to remove ligamentum flava at the cranial and caudal ends of the intended laminar expansion to facilitate opening the lamina. The laminae were then lifted carefully to prevent hinge breakage and expand the spinal canal diameter. The appropriate-sized Centerpiece laminoplasty plate (Medtronic Sofamor Danek) was placed at each level secured by a single screw onto the lamina and two screws at the level of the lateral masses. The excised spinous process mixed with artificial bone was used for bone grafting on the hinge side. The surgical wound was closed in layers after all the cervical levels had a laminoplasty plate. Patients were asked to wear a collar for 4–6 weeks postoperatively. All operations were performed by the same surgeon.

### Radiological parameters

**Figure 2:**
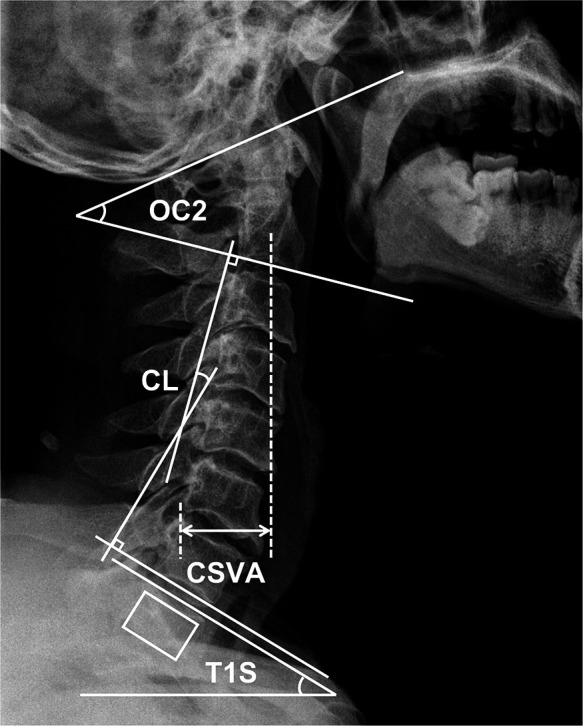
Measurements of cervical sagittal parameters utilized in this study.

A standing neutral lateral radiograph of the cervical and the global spine was obtained with patients facing forward and in a horizontal gaze (defined as −10° ≤ chin-brow to vertical angle ≤10° (10) before surgery and at the last follow-up. Radiological parameters measured included: occiput-C2 lordosis (OC2, the angle between the McGregor line and the inferior endplate of the C2), cervical lordosis (CL, the angle between the inferior endplate of C2 and the inferior endplate of C7), T1 slope (T1S, the angle between a horizontal line and the superior endplate of T1), CSVA (the distance from the posterior, superior corner of C7 to the plumbline from the centroid of C2). To patients with invisible T1S on the cervical radiography, the value of superior C7 slope was utilized to substitute for T1S (11, 12). Cervical parameters were measured using neutral lateral cervical x-rays. Changes of parameters were calculated as final follow-up data minus preoperative data. All the radiographic evaluations were completed by 2 independent spine surgeons who were not involved in the program. Measurements of sagittal parameters are illustrated in Figure 2.

### Clinical parameters

Japanese Orthopedic Association (JOA) score and neck disability index (NDI) were performed to assessment health-related quality of life (HRQOL) (13, 14). The JOA recovery rate, calculated as (postoperative JOA score—preoperative JOA score)/(full score—preoperative JOA score) × 100%, was used to evaluate the improvement of cervical neurological function. A JOA recovery rate of 100% indicated being cured; >60% indicated significantly effective; 25%–60% indicated effective; <25% indicated ineffective. An NDI <10% indicated no disability; 10%–30% indicated mild disability; 30%–50% indicated moderate disability; 50%–70% indicated severe disability; >70% indicated complete disability. Preoperative data were obtained from the medical records. Postoperative data were collected from outpatient follow-up records.

### Statistical analysis

All data were analyzed using SPSS Statistics (version 26.0, IBM Corp., Armonk, NY, USA). Continuous variables were compared between groups using the independent-samples *t*-test, Mann-Whitney *U* test, and paired-sample *t*-test. The chi-square test was used to compare composition ratios. Statistical significance was set at a level of *P* < 0.05. The results were presented as mean value ± standard deviation.

## Results

A total of 81 patients (48 males and 33 females, average age 64.69 ± 9.73 years) with a 17.88 ± 6.43 months follow-up were included. Table 1 summarizes cervical radiological and clinical parameters changes between the preoperative period and final follow-up. OC2 increased from 24.62 ± 6.92° to 27.63 ± 7.49°. CL decreased from 14.00 ± 8.59° to 10.30 ± 8.38°. Patients benefited from EOLP with an increase in JOA score and a decrease in NDI.

**Table 1:** Changes of radiological parameters and clinical parameters between preoperative period and final follow-up period.

Parameters	Preoperative (*n* = 81)	Final follow-up (*n* = 81)	*P*
OC2 (°)	24.62 ± 6.92	27.63 ± 7.49	0.000*
CL (°)	14.00 ± 8.59	10.30 ± 8.38	0.000*
T1S (°)	24.24 ± 6.18	24.17 ± 7.07	0.928
CSVA (mm)	23.25 ± 12.12	24.35 ± 12.59	0.425
JOA score	12.00 ± 1.88	14.72 ± 1.20	0.000*
NDI (%)	30.43 ± 18.44	13.80 ± 10.04	0.000*

OC2, occiput-C2 lordosis; CL, cervical lordosis; T1S, T1 slope; CSVA, cervical sagittal vertical axis; JOA, Japanese orthopedic association; NDI, neck disability index.

*P < 0.01.

To investigate the influence of preoperative T1S on clinical outcomes, patients were grouped according to the median preoperative T1S. Mean age of the low T1S group was younger than that of the high T1S group. Radiological parameters in terms of CL, T1S, and CSVA were significant greater in the high T1S group, while changes in these three parameters after cervical laminoplasty showed no difference in statistics. Concerning the clinical parameters, the final JOA score, JOA recovery rate, and final NDI were similar between groups (Table 2).

**Table 2:** Comparison of radiological parameters and clinical parameters between the low T1S group and the high T1S group.

Parameters	Low T1S (*n* = 40)	High T1S (*n* = 41)	*P*
Demographic parameters			
Age (years)	61.80 ± 8.61	68.81 ± 9.93	0.010*
Follow-up (months)	17.53 ± 6.30	18.38 ± 6.73	0.648
Operation level			0.696
C3–6	9	12	
C4–7	13	14	
C3–7	18	15	
Radiological parameters			
Pre-op OC2 (°)	25.99 ± 6.78	22.67 ± 6.79	0.093
ΔOC2 (°)	0.14 ± 0.24	0.16 ± 0.22	0.794
Pre-op CL (°)	11.60 ± 7.98	17.44 ± 8.43	0.015*
ΔCL (°)	−3.82 ± 5.74	−3.53 ± 4.11	0.844
Pre-op T1S (°)	20.17 ± 3.91	30.06 ± 3.60	0.000**
ΔT1S (°)	−1.98 ± 5.76	−0.46 ± 4.61	0.320
Pre-op CSVA (mm)	20.43 ± 8.99	27.28 ± 14.87	0.046*
ΔCSVA (mm)	1.61 ± 10.56	0.37 ± 8.70	0.658
Clinical parameters			
Pre-op JOA score	12.00 ± 2.00	12.00 ± 1.73	1.000
Final JOA score	14.70 ± 1.32	14.76 ± 1.04	0.858
JOA recovery rate (%)	52.78 ± 24.78	53.98 ± 17.43	0.849
Pre-op NDI (%)	30.93 ± 16.66	29.71 ± 21.14	0.819
Final NDI (%)	14.60 ± 9.81	12.67 ± 10.49	0.504

OC2, occiput-C2 lordosis; CL, cervical lordosis; T1S, T1 slope; CSVA, cervical sagittal vertical axis; JOA, Japanese orthopedic association; NDI, neck disability index.

*P < 0.05.

**P < 0.01.

Since the preoperative T1S did not make an influence on clinical outcomes of patients with CSM based on our data, patients were regrouped by the change of CL: patients with decreased CL postoperatively belonged to the CL loss group, patients with unchanged or increased CL belonged to the CL sustained group. Compared with that in the CL loss group, the final JOA score and JOA recovery rate were statistically greater, the final NDI was lower in the CL sustained group. Moreover, though there was no significant difference in preoperative T1S between the groups, T1S decreased significantly in the CL loss group (Table 3).

**Table 3:** Comparison of radiological parameters and clinical parameters between the CL sustained group and the CL loss group.

Parameters	CL sustained (*n* = 35)	CL loss (*n* = 46)	*P*
Demographic parameters			
Age (years)	63.75 ± 9.67	65.29 ± 9.87	0.586
Follow-up (months)	16.10 ± 5.25	19.03 ± 6.92	0.112
Operation level			0.563
C3–6	11	10	
C4–7	10	17	
C3–7	14	19	
Radiological parameters			
Pre-op OC2 (°)	24.43 ± 7.18	24.74 ± 6.87	0.875
ΔOC2 (°)	0.07 ± 0.21	0.19 ± 0.23	0.065
Pre-op CL (°)	12.92 ± 7.52	14.70 ± 9.26	0.473
ΔCL (°)	0.17 ± 1.58	−6.20 ± 5.01	0.000**
Pre-op T1S (°)	24.97 ± 6.80	23.77 ± 5.82	0.505
ΔT1S (°)	1.00 ± 4.05	−2.88 ± 5.54	0.010*
Pre-op CSVA (mm)	24.65 ± 11.25	22.35 ± 12.75	0.515
ΔCSVA (mm)	−0.55 ± 9.93	2.16 ± 9.67	0.337
Clinical parameters			
Pre-op JOA score	12.55 ± 1.73	11.64 ± 1.91	0.093
Final JOA score	15.45 ± 0.89	14.26 ± 1.15	0.000**
JOA recovery rate (%)	63.14 ± 23.82	46.91 ± 18.19	0.008**
Pre-op NDI (%)	30.30 ± 19.28	30.52 ± 18.20	0.968
Final NDI (%)	9.90 ± 8.30	16.32 ± 10.38	0.024*

OC2, occiput-C2 lordosis; CL, cervical lordosis; T1S, T1 slope; CSVA, cervical sagittal vertical axis; JOA, Japanese Orthopedic Association; NDI, neck disability index.

*P < 0.05.

**P < 0.01.

We hypothesized that the reduction of T1S in the CL loss group might affect the clinical outcomes. Thus, patients with postoperative LCL were further divided into two subgroups according to whether T1S decreased. Most notably, the T1S decreased subgroup had greater final JOA score and JOA recovery rate in statistics than the T1S sustained subgroup. CSVA tended to increase in the T1S sustained subgroup, while it reduced significantly in the T1S decreased subgroup (Table 4).

**Table 4:** Comparison of radiological parameters and clinical parameters between the T1S sustained subgroup and the T1S decreased subgroup.

Parameters	T1S sustained (*n* = 21)	T1S decreased (*n* = 25)	*P*
Demographic parameters			
Age (years)	65.00 ± 10.70	65.50 ± 9.54	0.892
Follow-up (months)	18.92 ± 6.73	19.11 ± 7.25	0.942
Operation level			0.911
C3–6	5	5	
C4–7	8	9	
C3–7	8	11	
Radiological parameters			
Pre-op OC2 (°)	23.35 ± 8.36	25.76 ± 5.60	0.343
ΔOC2 (°)	0.14 ± 0.26	0.24 ± 0.21	0.287
Pre-op CL (°)	18.25 ± 11.54	12.14 ± 6.49	0.069
ΔCL (°)	−6.45 ± 3.84	−6.03 ± 5.82	0.823
Pre-op T1S (°)	26.45 ± 4.25	21.83 ± 6.12	0.026*
ΔT1S (°)	1.98 ± 3.58	−6.39 ± 3.75	0.000**
Pre-op CSVA (mm)	21.98 ± 12.41	22.63 ± 13.34	0.891
ΔCSVA (mm)	7.82 ± 8.73	−1.92 ± 8.32	0.004**
Clinical parameters			
Pre-op JOA score	11.77 ± 1.69	11.56 ± 2.09	0.764
Final JOA score	13.77 ± 0.93	14.61 ± 1.20	0.043*
JOA recovery rate (%)	36.91 ± 8.02	54.13 ± 20.17	0.003**
Pre-op NDI (%)	29.85 ± 19.07	31.00 ± 18.09	0.865
Final NDI (%)	16.46 ± 11.20	16.22 ± 10.08	0.951

OC2, occiput-C2 lordosis; CL, cervical lordosis; T1S, T1 slope; CSVA, cervical sagittal vertical axis; JOA, Japanese orthopedic association; NDI, neck disability index.

*P < 0.05.

**P < 0.01.

## Discussion

Posterior laminoplasty generates an indirect decompression effect resulting from the posterior shift of the spinal cord from the anterior compressive lesions. This procedure successfully manages patients with CSM. Previous studies demonstrated that patients could achieve acceptable recovery of neurological function after posterior laminoplasty (15, 16). Nevertheless, there remain potential postoperative complications. Because of the destruction of the facet joint or damage to the paravertebral muscles and their attachments to the spinous processes, the cervical spine might show loss of lordosis and a tendency to tilt forward (17, 18). Diminished lordosis may elevate spinal intramedullary pressure and affect neurological function recovery (19). In the present study, LCL occurred after surgery in 46 (56.8%) patients.

Many previous studies explored the relationship between preoperative sagittal parameters and outcomes after cervical laminoplasty. Rao et al. reported that T1S-CL mismatching (T1S-CL > 20°) predicted worse postoperative NDI and JOA recovery rate in patients with CSM who underwent EOLP (20). Furthermore, Oshima et al. showd CSM patients with preoperative SVA > 50 mm had lower clinical outcome scores after cervical laminoplasty (21). Nori et al. also demonstrated C7 slope ≥30° correlated to lower postoperative JOA score and JOA recovery rate (22). Among all the sagittal parameters, T1S is closely associated with the shape of the cervical spine pre- and postoperatively (23, 24). Zhang et al. demonstrated that preoperative T1S was significantly correlated with LCL after laminoplasty in patients with CSM (25). Pan et al. showed that CSM patients with lower preoperative T1S had less neck pain during postoperative follow-up (17). In the present study, although there were differences in the preoperative cervical spine alignment between the lower T1S group and the greater T1S group, there were no significant variations in the JOA score, JOA recovery rate, or NDI at final follow-up (Table 2). Consistent with our research, Cho et al. showed that VAS, JOA score, NDI, and SF-36 at final follow-up were not affected by preoperative T1S in patients with CSM who underwent laminoplasty (26). The thoracolumbar sagittal balance influences T1S, and it changes reciprocally with the variation of spinal sagittal alignment. Hence, univariate analysis of the correlation between preoperative T1S and final clinical outcomes may be biased.

As mentioned previously, postoperative LCL after cervical laminoplasty is a common phenomenon which might exert a negative impact on clinical outcomes (5). Patients were divided into the CL sustained group and the CL loss group, and radiological/clinical parameters were compared between groups (Table 3). LCL contributes to progressive kyphotic alignment change, leading to postoperative residual anterior compression and worse outcomes at long-term follow-up (27). Consistently, the CL loss group had a lower final JOA score and JOA recovery rate, but higher final NDI at the final follow-up in our study. Similarly, Xu et al. found that postoperative LCL indicated worse JOA score and JOA recovery rate in laminoplasty treated patients (28). Moreover, we also found that T1S decreased significantly after surgery in the CL loss group. T1S was positively correlated with CL, which means a greater T1S yielded a greater magnitude of CL in the asymptomatic population (10). Thus, we speculated that the decrease of T1S in the CL loss group was a compensatory mechanism of LCL after cervical laminoplasty to maintain an appropriate alignment.

It remains unclear whether the decrease of T1S improves outcomes in patients who undergo cervical laminoplasty. According to the change of T1S, the CL loss group was further divided into the T1S sustained and T1S decreased subgroups. Results illustrated there was no significant difference in preoperative cervical sagittal parameters and thoracolumbar sagittal parameters except for T1S between the two subgroups (Table 4). The T1S sustained subgroup had lower JOA scores and JOA recovery rate at the final follow-up. The variation in CSVA was positive and significantly higher in the T1S sustained group, which means the cervical spine tended to tilt forward. Smith et al. assessed 56 patients with CSM and reported that improved JOA score was negatively correlated with CSVA (29). In a study of 249 patients who underwent EOLP, Zhang et al. also showed that preoperative CSVA and postoperative CSVA were both associated with postoperative axial symptoms (30). Larger CSVA correlated with higher intramedullary cord pressure, which results in histologic changes in the spinal cord and deterioration of neurological function (19, 31). The reduction of T1S improved neurological function recovery after cervical laminoplasty by regulating CSVA.

OC2 (a description of upper cervical shape) is measured by the angle subtended by the McGregor line of sight and a parallel line along the inferior endplate of C2. Previous studies demonstrated that OC2 and CL work inversely. Loss of lordosis in the subaxial cervical spine can be compensated for by the hyperlordotic upper cervical spine (8, 32, 33). In our study, the increase of OC2 compensated for LCL in patients who underwent cervical laminoplasty (Table 1). Nevertheless, there were no significant differences in preoperative OC2 or change of OC2 between the CL sustained group and the CL loss group. These findings suggest that decreased T1S is the primary compensatory mechanism of CL loss and contributes to postoperative function recovery, while the increase of OC2 might be responsible for maintaining horizontal gaze.

There are still several limitations in this study. First, because of the study’s retrospective nature, only the data contained in the medical records could be analyzed. Second, the sample size was relatively small and from a single center. Third, postoperative thoracolumbar parameters, which could influence the change of T1S, were not included. Prospective and well-designed studies will be necessary to identify the compensatory mechanisms associated with postoperative neurological function recovery.

### Patient presentation

**Figure 3:**
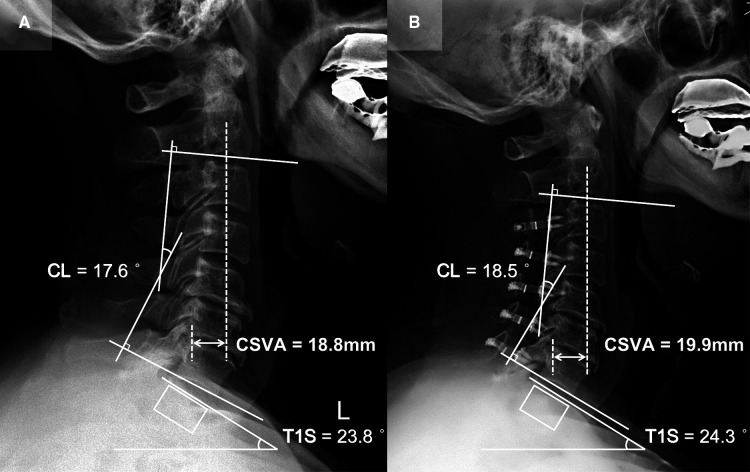
Patient case of the CL sustained group. (**A**) Preoperative lateral cervical radiograph (CL = 17.6°, T1S = 23.8°, CSVA = 18.8 mm). (**B**) Lateral cervical radiograph at final follow-up (15 months after surgery, CL = 18.5°, T1S = 24.3°, CSVA = 19.9 mm).

Patient 1 (CL sustained group; Figure 3): A 65-year-old male with a 15-month follow-up. The preoperative CL was 17.6°, the preoperative T1S was 23.8°. Preoperative JOA score and NDI were 12 and 12%, respectively. CL and T1S were 18.5° and 24.3° at final follow-up, respectively. The change of CSVA was +1.1 mm. JOA score increased from 12 to 16, while NDI decreased from 12% to 6%. The JOA recovery rate was 80%.

**Figure 4:**
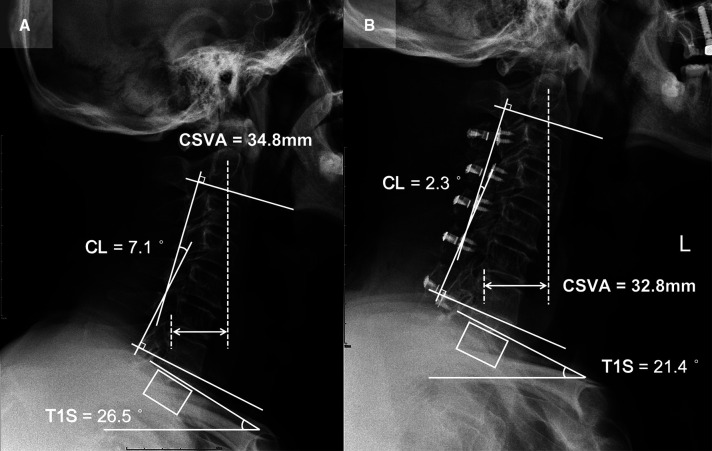
Patient case of the CL loss group, T1S decreased subgroup. (**A**) Preoperative lateral cervical radiograph (CL = 7.1°, T1S = 26.5°, CSVA = 34.8 mm). (**B**) Lateral cervical radiograph at final follow-up (15 months after surgery, CL = 2.3°, T1S = 21.4°, CSVA = 32.8 mm).

Patient 2 (CL loss group, T1S decreased subgroup; Figure 4): A 65-year-old male with a 15-month follow-up. The preoperative CL was 7.1°, the preoperative T1S was 26.5°. Preoperative JOA score and NDI were 12 and 10%, respectively. CL and T1S were 2.3° and 21.4° at final follow-up, respectively. The change of CSVA was −2 mm. JOA score increased from 12 to 15, while NDI decreased from 10% to 6%. The JOA recovery rate was 60%.

**Figure 5:**
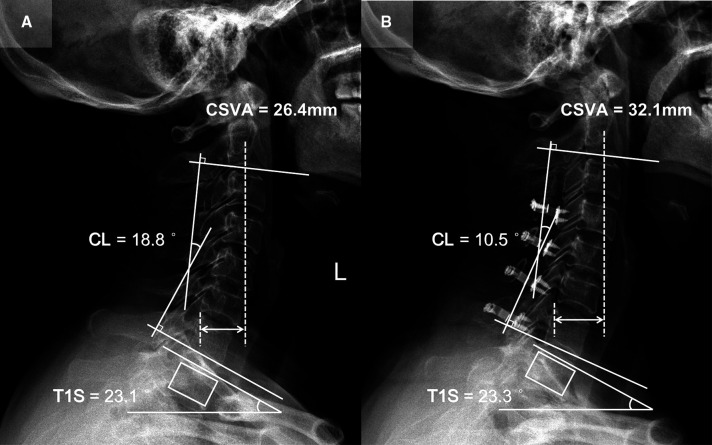
Patient case of the CL loss group, T1S sustained subgroup. (**A**) Preoperative lateral cervical radiograph (CL = 18.8°, T1S = 23.1°, CSVA = 26.4 mm). (**B**) Lateral cervical radiograph at final follow-up (14 months after surgery, CL = 10.5°, T1S = 23.3°, CSVA = 32.1 mm).

Patient 3 (CL loss group, T1S sustained subgroup; Figure 5): A 56-year-old female with a 14-month follow-up. The preoperative CL was 18.8°, the preoperative T1S was 23.1°. Preoperative JOA score and NDI were 11 and 30%, respectively. CL and T1S were 10.5° and 23.3° at final follow-up, respectively. The change of CSVA was +5.7 mm. JOA score increased from 11 to 14, while NDI decreased from 30% to 14%. The JOA recovery rate was 50%.

## Conclusion

The decrease of T1S is a compensatory mechanism of LCL in patients who undergo cervical laminoplasty. Decreased T1S prevents the tendency of the cervical spine to tilt forward by regulating CSVA, which facilitates the recovery of neurological function postoperatively.

## Data Availability

The original contributions presented in the study are included in the article/Supplementary Material, further inquiries can be directed to the corresponding author/s.
